# Computational cell model based on autonomous cell movement regulated by cell-cell signalling successfully recapitulates the "inside and outside" pattern of cell sorting

**DOI:** 10.1186/1752-0509-1-43

**Published:** 2007-09-20

**Authors:** Takuya T Maeda, Itsuki Ajioka, Kazunori Nakajima

**Affiliations:** 1 Department of Anatomy, Keio University School of Medicine, 35 Shinanomachi, Shinjuku-ku, Tokyo 160-8582, Japan; 2 Department of Molecular Neurobiology, Institute of DNA Medicine, Jikei University School of Medicine, 3-25-8, Nishi-shinbashi, Minato-ku, Tokyo 105-8461, Japan; 3 Computational and Experimental Systems Biology Group, RIKEN Genomic Sciences Center, 1-7-22 Suehiro-cho, Tsurumi-ku, Yokohama, Japan; 4 Department of Developmental Neurobiology, St. Jude Children's Research Hospital, 332N Lauderdale, Memphis, TN 38105, USA

## Abstract

**Background:**

Development of multicellular organisms proceeds from a single fertilized egg as the combined effect of countless numbers of cellular interactions among highly dynamic cells. Since at least a reminiscent pattern of morphogenesis can be recapitulated in a reproducible manner in reaggregation cultures of dissociated embryonic cells, which is known as cell sorting, the cells themselves must possess some autonomous cell behaviors that assure specific and reproducible self-organization. Understanding of this self-organized dynamics of heterogeneous cell population seems to require some novel approaches so that the approaches bridge a gap between molecular events and morphogenesis in developmental and cell biology. A conceptual cell model in a computer may answer that purpose. We constructed a dynamical cell model based on autonomous cell behaviors, including cell shape, growth, division, adhesion, transformation, and motility as well as cell-cell signaling. The model gives some insights about what cellular behaviors make an appropriate global pattern of the cell population.

**Results:**

We applied the model to "inside and outside" pattern of cell-sorting, in which two different embryonic cell types within a randomly mixed aggregate are sorted so that one cell type tends to gather in the central region of the aggregate and the other cell type surrounds the first cell type. Our model can modify the above cell behaviors by varying parameters related to them. We explored various parameter sets with which the "inside and outside" pattern could be achieved. The simulation results suggested that direction of cell movement responding to its neighborhood and the cell's mobility are important for this specific rearrangement.

**Conclusion:**

We constructed an *in silico *cell model that mimics autonomous cell behaviors and applied it to cell sorting, which is a simple and appropriate phenomenon exhibiting self-organization of cell population. The model could predict directional cell movement and its mobility are important in the "inside and outside" pattern of cell sorting. Those behaviors are altered by signal molecules and consequently affect the global pattern of the cell sorting. Our model is also applicable to other developmental processes beyond cell sorting.

## Background

The development of multicellular organisms is highly organized in a complex spatio-temporal manner that enables an enormous number of cells to form a single individual autonomously. These programmed processes seem miraculous, considering that they occur in an extremely reproducible manner that extends beyond individuals and generations. Interestingly, certain patterns that are at least reminiscent of morphogenesis are known to be recapitulated in reaggregation cultures of dissociated cells from embryonic tissues. For example, embryonic cells dissociated from two different tissues will sort into two separate regions when cultured as a randomly mixed aggregate of cells. This rearrangement of the cells proceeds according to the cell's tissue origins and is reminiscent of the original embryonic structures [1,2]. That is, randomly mixed cells seem to reconstruct their original cellular arrangement in response to their surroundings. Thus, cell-sorting may provide insights into the mechanism by which embryonic cells undergo self-organization.

To clarify which cell characteristics are important for the highly reproducible self-organization of cells into tissues, we focused here on a certain cell-sorting in a simple heterogeneous population of two cell types – namely, the accumulation of one cell type in the central region of a randomly mixed aggregate of two different embryonic cell types and the accumulation of the second cell type in the area surrounding the first cell type (an "inside and outside" cell-sorting pattern). The "inside and outside" pattern is important because this pattern reflects an original embryonic structure [1].

For the process of cell-sorting within the aggregate, adhesion properties of cells are considered as an important factor. So far, two major hypotheses have been proposed: the "Specific Adhesion Hypothesis (SAH)" and the "Differential Adhesion Hypothesis (DAH)". The SAH explains cell-sorting using the concept of selective affinity of cell adhesion; in other words, cells of the same type gather by adhering to each other and, conversely, the population of a specific type of cells excludes different cell types by not adhering to them well [3]. This adhesive relationship predicts that homophilic adhesions of both cell types are stronger than heterophilic adhesions, as observed in many experiments [4-8]. However, the SAH could not reasonably explain the "inside and outside" configuration of cell-sorting [9].

The DAH defines *adhesion energy *as a physical quantity and explains cell-sorting using a physical law involving the *free energy minimum *or *adhesion energy maximum*; that is, the most energetically stable structure is formed over time [10]. Based on the DAH, several theoretical models have been proposed. Although the DAH predicts the "inside and outside" pattern in a certain adhesive relationship (described in the Discussion), some models did not reproduce the "inside and outside" pattern [11-15] and other models required additional assumptions, such as remote interactions among cells [16-19] because of the existence of cellular configurations that had local maximums in the total adhesion energy. Cell rearrangements were halted at a local maximum in the energy landscape.

Remote cellular interactions are essential for certain types of cell-sorting. In the slime mold *Dictyostelium discoideum*, excitable cells move in response to a diffusive chemoattractant cAMP emanated from pacemaker cells; that is, the pacemaker cells operate remote excitable cells via diffusive signals. Cell-sorting in the slime mold *D. discoideum *has been vigorously studied. In model systems for the pattern formation in *D. discoideum*, not only cell-sorting but also collective cell movements have been successfully described in computational experiments [20-22] and mathematical analysis [21]. On the other hand, remote cellular interactions during the process of cell-sorting within the randomly mixed aggregates of embryonic cells remains unknown. Thus, we did not assume remote cellular interactions in this study.

The above models of cell-sorting for randomly mixed aggregates of embryonic cells were lattice models in which a cell occupies one lattice in a finite lattice space and moves by changing its position to a neighboring lattice. Thus, the cell movements were discrete. Glazer and Graner (1992) successfully reproduced the "inside and outside" configuration of cell-sorting by constructing a model in which cell movement was quasi-continuous [23,24]. Models of cell-sorting with continuous cell movement have also been proposed [25,26].

However, when the cellular events in developing tissues are observed *in vivo*, the involvement of other aspects of cellular behavior in pattern formation becomes apparent. During sea urchin gastrulation, for example, the primary mesenchymal cells migrate to prospective ventrolateral regions of the blastocoel and fuse into syncytial cables [27,28]. Convergent extension is a cooperative cellular behavior that is necessary for archenteron invagination [29]. In addition to this cooperative behavior, secondary mesenchymal cells also assist during archenteron invagination [30,31]. In avian embryos, the trunk neural crest cells journey long distances to their final destinations via different pathways, depending on their region of origin in the neural crest, and differentiate into appropriate cell types at their final destinations [32-36]. In mammals, the cerebral cortex arises from the migration of huge numbers of neurons. These neurons form distinct cortical layers, depending on their birthdates [37], and function in a layer-specific manner. These examples indicate that each cell behaves individually during development, receiving signals from its surroundings and varying its inner state according to those signals, thereby exerting an effect on its surroundings either individually or cooperatively or moving autonomously or en masse. In this manner, the cell finally arrives at its proper position and begins to function in an appropriate manner. Such cellular behaviors are essential for development.

Based on these observations and consideration, we constructed an *in silico *cell model with a flexible shape, directional motility, and sensitivity to parameters like autonomous cellular behaviors; we then used this model to examine what differences in cellular behavior parameters would lead to the reproducible generation of an "inside and outside" pattern of cell-sorting. In this manner, we successfully recapitulated the specific rearrangement of cells observed during "inside and outside" cell-sorting by inputting data reflecting a randomly mixed aggregate of two different cell types. The results of these computer simulations suggested that the directional movement of the cells and the cell's mobility were important for this specific rearrangement.

## Results

### Outline of *in silico *cell model

**Figure 1:**
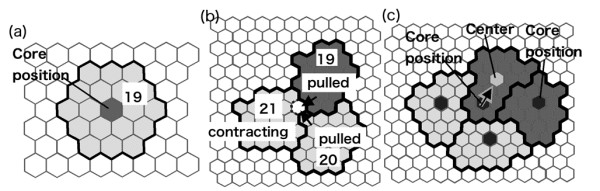
**Modeling of cells**. (a) A single cell with a size of 19 hexagons and a nearly round shape in a free lattice space. (b) A cell with a larger (21 hexagons) than standard (19 hexagons) size contracts with two other cells and "pulls" either of the two cells, which is "stretched" by cell adhesion. (c) A distorted cell shifts its core position in the direction indicated by the black arrow because its core position is far from the center of the cell's body.

We explain our model briefly here and give a full and particular account of the model in Methods section. Our model is on two-dimensional hexagonal lattice space. Each cell occupies multiple hexagons in the lattice space (Fig. 1a), with the number of hexagons occupied by a single cell representing the cell's size. For dynamic change in the cell's shape in response to its simulated surroundings, cell size is variable about its standard size that is set in a simulation. Each cell contains one hexagon that the cell never loses; this hexagon represents the cell's "core position." that is regarded as the position at which a cell tries to remain in place at any given time point. This core position is used for modeling purpose only and does not represent a specific biological entity.

If a cell is smaller than the standard cell size, the cell attempts to occupy a hexagon nearest to its core position. If a cell is larger than the standard size, the cell abandons the hexagon furthest from its core position. Iteration of these procedures makes a single cell in a free space round (Fig. 1a). When cells are put as a cluster, an expanding cell in the cluster causes positional conflict between its neighboring cell, and a contracting cell pulls its neighboring cell via cell adhesion (Fig. 1b). Because of these spatial interactions, each cell is often distorted, and the core position of the cell is not always at its center. If the cell shape is far from round, the core position is shifted to an adjacent hexagon closer to the cell's center (Fig. 1c). This shift represents a cell being pushed away from its desired position.

**Figure 2:**
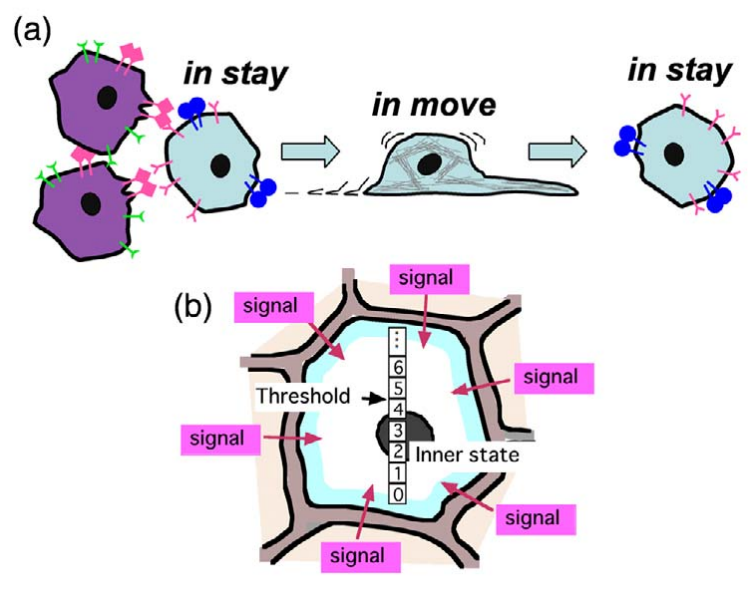
**Concept of autonomous cell movement**. (a) Each cell has two states, *in stay *and *in move*, for cell movement. A cell *in stay *is activated by signals from different cell type, and begins to move, changing its state into *in move*-state. The cell *in move *continues to move for short periods and then ceases from moving and returns to *in stay*. (b) Idea of activation mechanism: The signals from different cell types increase an inner state of a cell *in stay*. If the inner state of the cell exceeds a threshold, the cell *in stay *is activated and becomes *in move*-state.

In addition to the spatial mechanical interaction between cells, each cell receives signals from neighboring cells. Here, we postulate the signals trigger autonomous directional cell movement. Each cell has two states, *in stay *and *in move*, for cell movement. A cell *in stay *receives signals from neighboring cells (Fig. 2a). The signals from different cell types increase an inner state of the cell *in stay*, while those from the same cell type as the cell in question do not (Fig. 2b). If the inner state of the cell exceeds a threshold, the cell *in stay *becomes activated and then begins to move, changing its *in stay*-state into *in move*-state. The cell *in move *continues to move for short periods without receiving signals from neighboring cells. After the short periods, the cell *in move *ceases from moving and returns to *in stay*.

### Parameters regulating cellular behaviors

**Figure 3:**
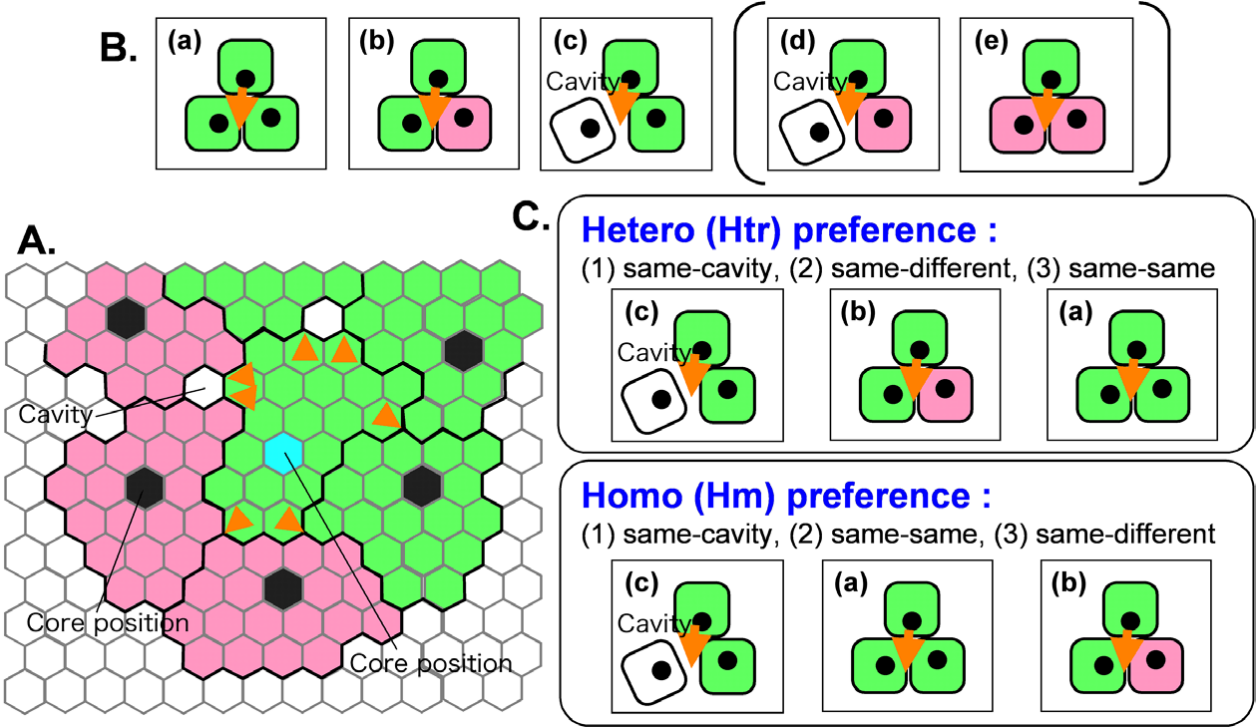
**Idea of directional cell movment**. A: A green cell with a cyan-colored core position tries to move. There are several gaps as indicated by orange arrowheads. B: There are five possible gaps. White cell means that both cell types are possible. C: Two different orders of preferred penetrable gaps.

Five parameters that regulate cell behaviors were defined in this cell model. We briefly explain these parameters here and give more details in Methods section. (1) MAXIMAL DISTORTION SCORE means what extent each cell resist pressure by neighboring cells. If a cell has a large value of MAXIMAL DISTORTION SCORE, the cell resists mechanical conflict from neighboring cells. (2) ACTIVATION THRESHOLD means insensitivity to signals from neighboring cells. If a cell has a small value of ACTIVATION THRESHOLD, the cell is easily activated by the signals from different cell types, and start moving. (3) DRAGGING TIME means period in which cell is *in move*-state after a single activation by signals from neighboring cells. (4) SINGLE MOVING DISTANCE means change in position in a single activation. (5) GAP PREFERENCE means orders of preferred penetrable gaps for cell movement. Cell movement is possible only for penetrating a gap between neighboring cells. Here, we consider three gaps as follows: gaps between 1) the same cell types, 2) the same cell type and different cell type, and 3) the same cell type and cavity (Fig. 3). Two different orders of preferred penetrable gaps are postulated. In the first situation (referred to as the Hetero preference [Htr]), the preferred gaps are selected in the following order: (1) gaps between a cell of the same type as the cell in question and a cavity, (2) gaps between a cell of same cell type as the cell in question and a cell of a different cell type, and (3) gaps between two cells of the same cell type as the cell in question. In the second situation (referred to as the Homo preference [Hm]), the preferred gaps are selected in the following order: (1) gaps between a cell of the same cell type as the cell in question and a cavity, (2) gaps between two cells of the same cell type as the cell in question, and (3) gaps between a cell of the same cell type as the cell in question and a cell of a different cell type. When gaps with the same priority are present, a gap to be penetrated is randomly selected. It is to note that the above-mentioned idea of activation mechanism (Fig. 2b) and both the Htr and Hm gap preferences commonly include specific adhesion properties.

### Simulation results of cell sorting

The MAXIMAL DISTORTION SCORE was set at 0.45. Several parameter sets in which ACTIVATION THRESHOLD, DRAGGING TIME, SINGLE MOVING DISTANCE, and GAP PREFERENCE were varied were then explored. To classify the aggregation patterns, we defined two indexes: CELL DISTRIBUTION RATIO (ratio of cells in the central region to those in the peripheral region) and PERIMETER RATIO. The definitions of them are described in Methods section. The "inside and outside" patterns in the following results were determined using these two indexes.

**Figure 4:**
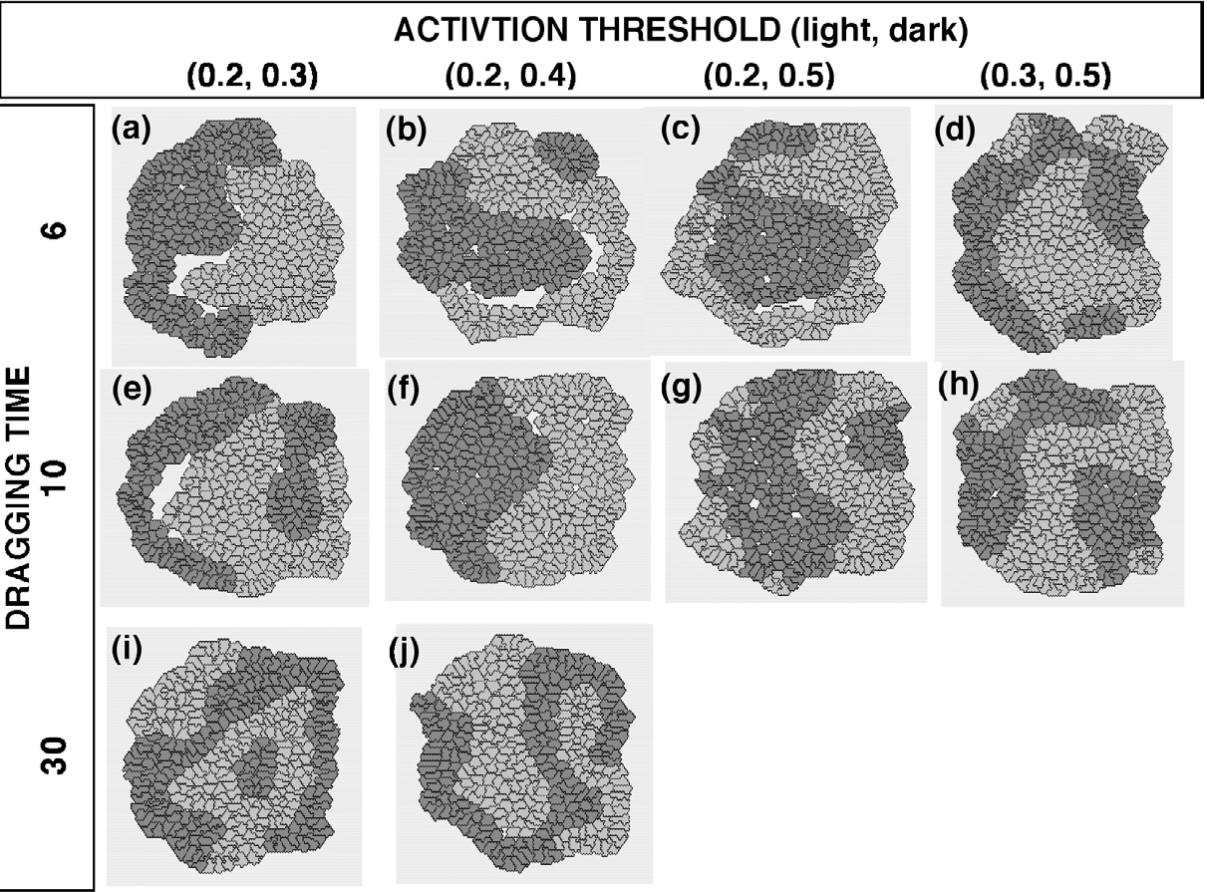
**Final configurations of aggregates of randomly mixed cells with different ACTIVATION THRESHOLDs and an Hm gap preference**. The following conditions were common to both cell types: DRAGGING TIME = {6, 10, or 30} SINGLE MOVING DISTANCE = 1; Hm preference. The areas of the inner cavities within the aggregates in (a), (b), (c), and (e) were 0.484, 0.428, 0.200, and 0.535, respectively; thus, all of these aggregates exhibited SSPs according to the definition described in the text. Although some aggregates occasionally exhibited an ''inside and outside'' pattern of cell-sorting, this pattern appeared only by chance and was not observed reproducibly.

**Figure 5:**
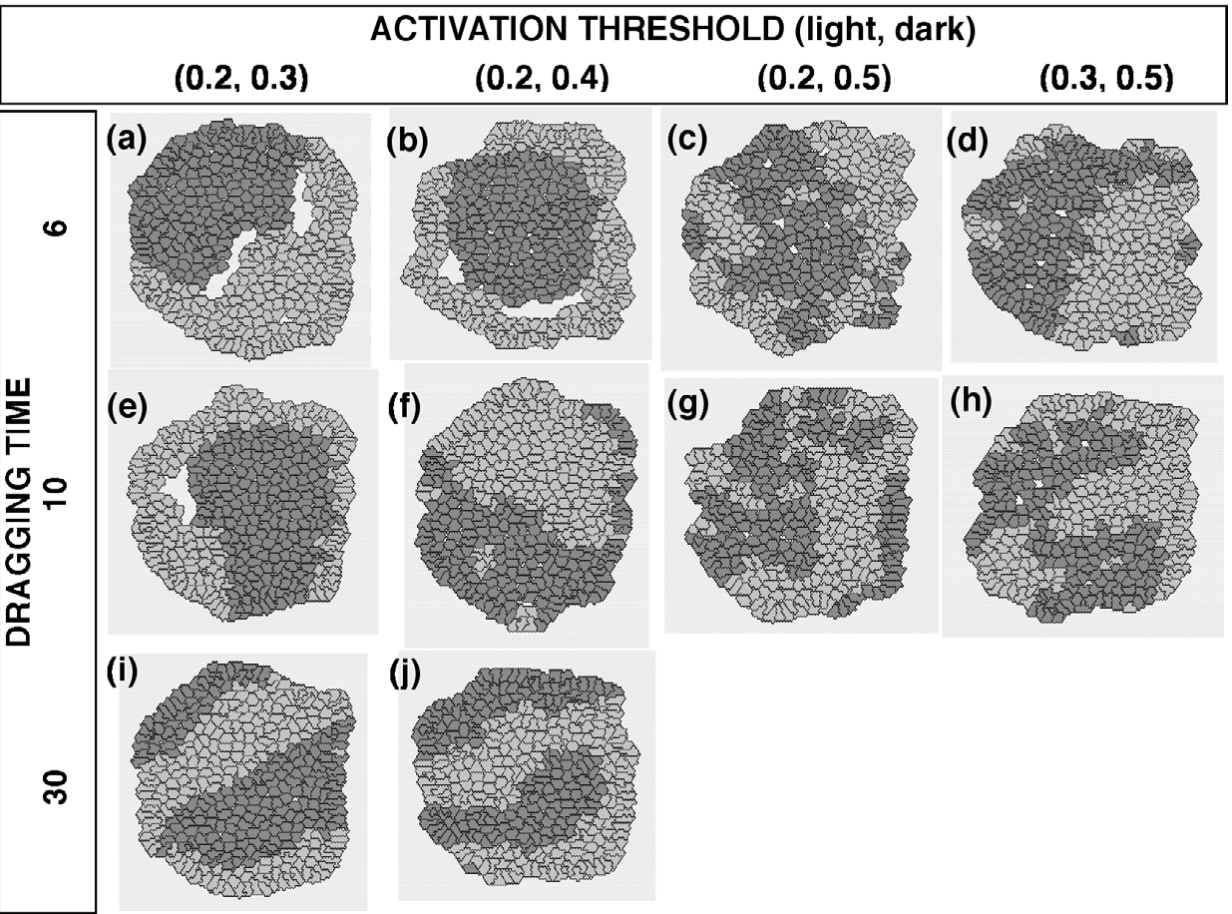
**Final configurations of aggregates of randomly mixed cells with different ACTIVATION THRESHOLDs and an Htr gap preference**. The common conditions to both cell types: DRAGGING TIME = {6, 10, or 30}; SINGLE MOVING DISTANCE = 1; Htr preference. The areas of the inner cavities within the aggregates in (a), (b), and (e) were 0.444, 0.482, and 0.424, respectively (SSPs).

In the first series of simulations, the two cell types were given different ACTIVATION THRESHOLD values but DRAGGING TIME, SINGLE MOVING DISTANCE and GAP PREFERENCE parameters were kept the same. Under these conditions, the two cell types segregated from each other within the aggregates during each simulation, but the "inside and outside" configuration – where one cell type forms a central cluster and the other cell type surrounds that cluster – was not reproducibly generated (Figs. 4, 5). When the ACTIVATION THRESHOLD and DRAGGING TIME parameters were set at relatively small values for either cell type, the cell type in question moved extensively and its cluster tended to detach temporarily from the cluster of the other cell type, generating a huge inner cavity within the aggregate. Once the cells reached their final configuration, the cell movements became relatively modest and the huge inner cavity tended to diminish and often vanished. The detachment of the two cell types from each other was inappropriate for the analysis of the cell-sorting mechanism that we wished to examine in this study. Thus, we defined this type of process as a "separated sorting process" (SSP) (Fig. 4a,b,c,e, Fig. 5a,b,e) and decided to exclude all cases with an SSP pattern from further quantitative analysis. To objectively determine whether SSP had occurred during a simulation, we measured the area of the inner cavity in the final configuration and set the threshold for the area of an inner cavity as 0.2. As shown in Table 1, SSP was observed for several parameter combinations in this study.

**Table 1:** Summary of cell arrangement patterns in the *in silico *aggregates

Pattern*	Cases**
Inappropriate because of separated sorting process (SSP)	Fig. 2a-c,e; Fig. 3a,b,e; Fig. 4a,b,g,h; Fig. 5a-c,e,f; Fig. 6a,b

**Figure 6:**
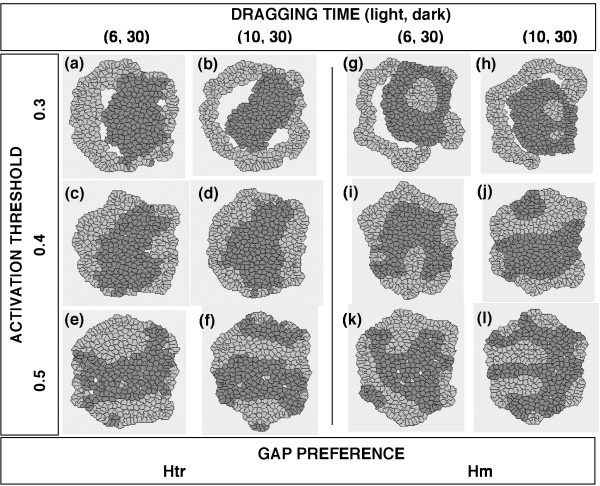
**Final configurations of aggregates of randomly mixed cells with different DRAGGING TIMEs**. The common conditions to both cell types: ACTIVATION THRESHOLD = {0.3, 0.4, or 0.5}; SINGLE MOVING DISTANCE = 1; GAP PREFERENCE = {Htr or Hm}. The areas of the inner cavities within the aggregates in (a), (b), (g), and (h) were 0.378, 1.973, 1.675, and 0.374, respectively (SSPs). The CELL DISTRIBUTION RATIOs of the dark cell type in (c) and (d) were 3.480 ± 0.757 (S.D.) and 3.629 ± 1.314, respectively. The PERIMETER RATIOs in (c) and (d) were 0.100 ± 0.042 and 0.087 ± 0.051, respectively. Thus, (c) and (d) represented an "inside and outside" pattern. The CELL DISTRIBUTION RATIOs and PERIMETER RATIOs of the other figures are shown in Table 1.

Inside and outside	Fig. 4c,d; Fig. 5d; Fig. 6c-e

Roughly inside and outside	Fig. 4i; Fig. 6f

Others	Fig. 2d: (0.774 ± 0.683, 3.059 ± 2.188) or (1.868 ± 1.095, 0.590 ± 0.517). Fig. 2f: (1.056 ± 0.143, 0.092 ± 0.270) or (0.872 ± 0.160, 1.057 ± 0.215). Fig. 2g: (0.684 ± 0.336, 2.203 ± 1.109) or (1.571 ± 0.616, 0.564 ± 0.302). Fig. 2h: (0.767 ± 0.320, 2.261 ± 1.564) or (1.710 ± 1.335, 0.585 ± 0.291). Fig. 2i: (1.364 ± 0.651, 1.295 ± 1.174) or (1.167 ± 0.687, 1.451 ± 0.996). Fig. 2j: (1.545 ± 0.757, 0.935 ± 0.624) or (0.899 ± 0.524, 1.454 ± 0.763). Fig. 3c: (0.552 ± 0.202, 1.751 ± 1.168) or (2.122 ± 1.046, 0.732 ± 0.337). Fig. 3d: (0.778 ± 0.416, 2.055 ± 1.429) or (1.676 ± 1.083, 0.632 ± 0.274). Fig. 3f: (0.622 ± 0.435, 1.954 ± 1.529) or (1.924 ± 1.064, 0.828 ± 0.559). Fig. 3g: (0.801 ± 0.109, 1.251 ± 0.523) or (1.396 ± 0.249, 0.908 ± 0.313). Fig. 3h: (0.775 ± 0.185, 1.692 ± 0.528) or (1.310 ± 0.281, 0.657 ± 0.263). Fig. 3i: (1.060 ± 0.927, 1.381 ± 0.609) or (1.313 ± 0.585, 0.997 ± 0.829). Fig. 3j: (0.976 ± 0.196, 1.060 ± 0.339) or (1.132 ± 0.256, 1.026 ± 0.318). Fig. 4e: (0.353 ± 0.121, 3.973 ± 1.259) or (2.516 ± 0.675, 0.268 ± 0.064). Fig. 4f: (0.521 ± 0.111, 2.444 ± 0.988) or (1.770 ± 0.434, 0.473 ± 0.193). Fig. 4j: (0.488 ± 0.281, 3.221 ± 1.328) or (2.248 ± 0.981, 0.369 ± 0.182). Fig. 4k: (0.345 ± 0.139, 4.957 ± 2.628) or (2.745 ± 0.820, 0.245 ± 0.103). Fig. 4l: (0.662 ± 0.165, 2.035 ± 0.817) or (1.366 ± 0.329, 0.563 ± 0.220). Fig. 5g: (0.374 ± 0.091, 3.650 ± 1.897) or (2.722 ± 1.498, 0.318 ± 0.111). Fig. 5h: (0.531 ± 0.159, 3.468 ± 1.903) or (2.220 ± 1.032, 0.360 ± 0.163).

* See methods for the criteria used to define of each pattern. ** Parentheses show (average of CELL DISTRIBUTION RATIO ± S.D., average of PERIMETER RATIO ± S.D.), N = 6. The former parentheses are for the light cell type and the latter are for the dark cell type.

In our next series of simulations, the two cell types were given different DRAGGING TIME values and the other parameters were kept the same. When GAP PREFERENCE of both cell types was set at Htr, the "inside and outside" pattern of cell sorting was reproducibly attained using certain parameter sets (Fig. 6c,d); however, an "inside and outside" configuration was not obtained when the GAP PREFERENCE of both cell types was set at Hm (Fig. 6g–l). In the "inside and outside" configurations, the cell type with a shorter DRAGGING TIME (shown as the light cells) always surrounded the cell type with a longer DRAGGING TIME (shown as the dark cells). When an ACTIVATION THRESHOLD = 0.3 was used, all the results contained SSPs (Fig. 6a,b,g,h).

**Figure 7:**
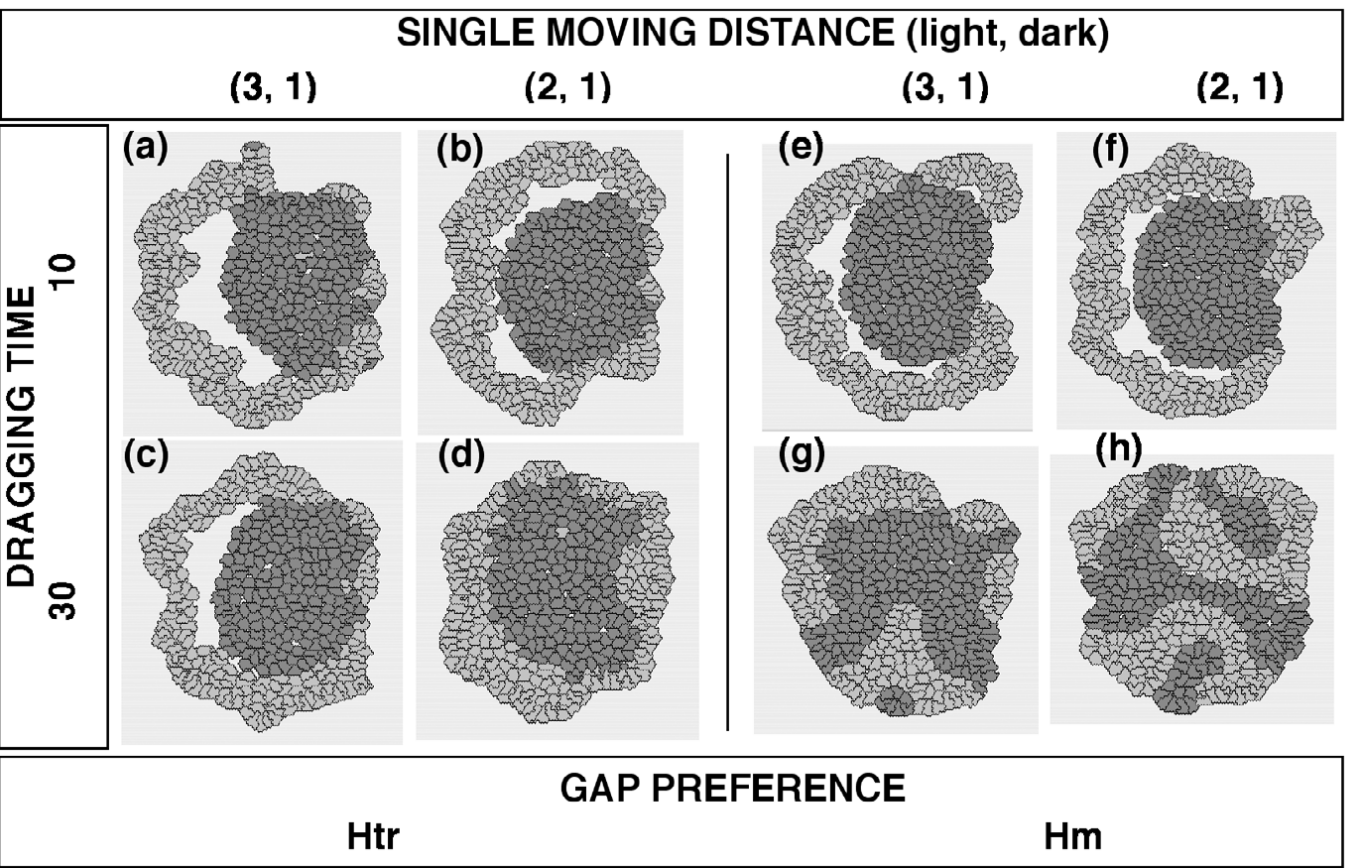
**Final configurations of aggregates of randomly mixed cells with different SINGLE MOVING DISTANCEs**. The common conditions to both cell types: ACTIVATION THRESHOLD = 0.4; DRAGGING TIME = {10 or 30}; GAP PREFERENCE = {Htr or Hm}. The areas of the inner cavities within the aggregates in (a), (b), (c), (e), and (f) were 2.597, 0.834, 1.564, 1.352, and 1.420, respectively (SSPs). The CELL DISTRIBUTION RATIO and PERIMETER RATIO of the dark cell type in (d) were 3.948 ± 1.611 (S.D.) and 0.099 ± 0.082, respectively.

When the cells were given different SINGLE MOVING DISTANCE values and the other parameters were kept the same, "inside and outside" cell-sorting was attained using certain parameter sets in which both cell types had their GAP PREFERENCE set at Htr (Fig. 7d). The cell type with a larger SINGLE MOVING DISTANCE (shown as the light cells) always surrounded the cell type with a shorter SINGLE MOVING DISTANCE (shown as the dark cells). When a DRAGGING TIME = 10 or a SINGLE MOVING DISTANCE = 3 was used, an SSP occurred (Fig. 7a,b,c,e,f).

**Figure 8:**
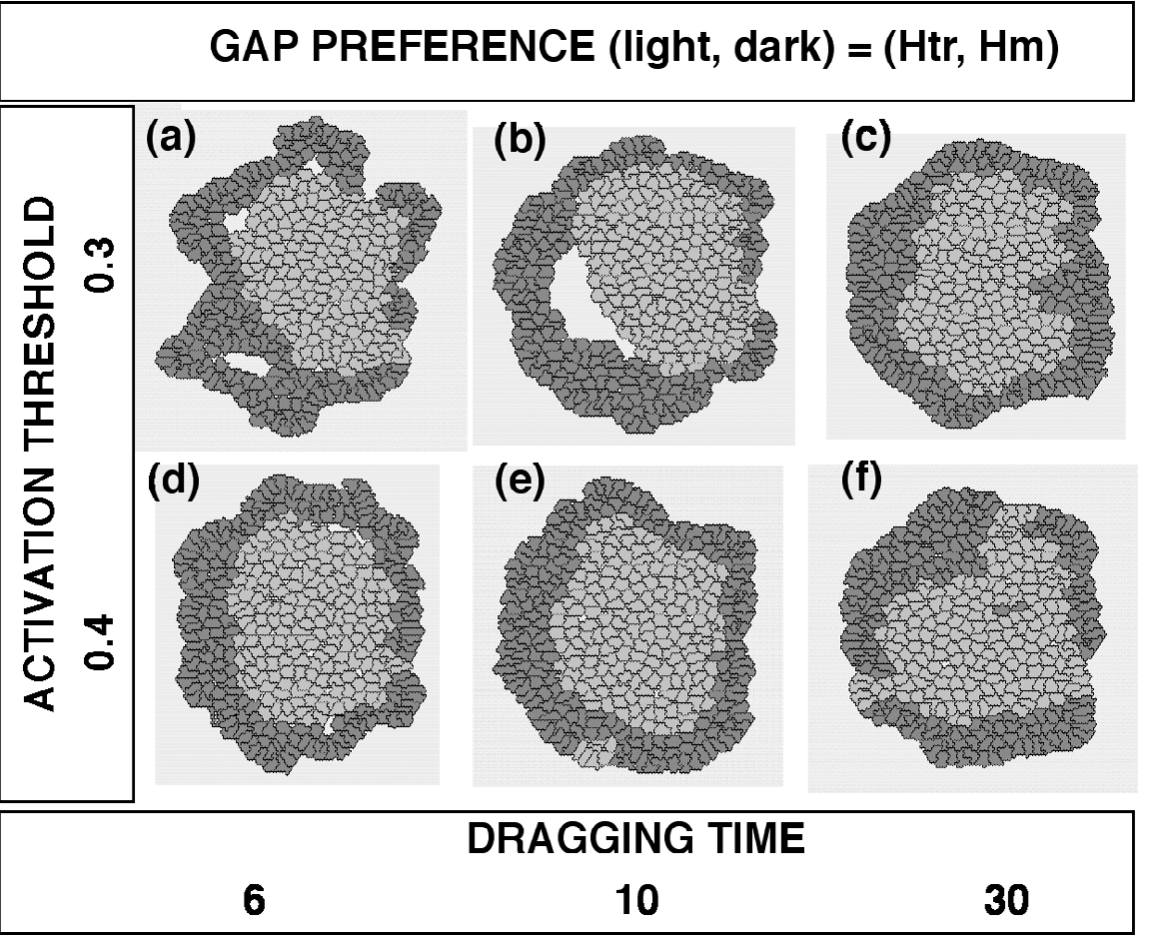
**Final configurations of aggregates of randomly mixed cells with different GAP PREFERENCE**. The common conditions to both cell types: ACTIVATION THRESHOLD = {0.3 or 0.4}; DRAGGING TIME = {6, 10, or 30}; SINGLE MOVING DISTANCE = 1. The areas of the inner cavities within the aggregates in (a) and (b) were 2.597 and 0.834, respectively (SSPs). The CELL DISTRIBUTION RATIOs of the light cell type in (c), (d) and (e) were 5.274 ± 2.402 (S.D.), 8.132 ± 3.406, and 5.326 ± 1.281, respectively. The PERIMETER RATIOs in (c), (d) and (e) were 0.077 ± 0.059, 0.061 ± 0.056 and 0.077 ± 0.058, respectively.

Although the Hm preference did not seem to contribute to the "inside and outside" configuration under the above-mentioned conditions, "inside and outside" cell sorting occurred when one cell type had an Hm preference and the other cell type had an Htr preference (Fig. 8c–e). The cell type with the Htr preference (shown as the light cells) was always located near the center of the aggregate and was surrounded by the cell type with the Hm preference (shown as the dark cells). When an ACTIVATION THRESHOLD = 0.3 and a DRAGGING TIME ≤ 10 were used, an SSP occurred (Fig. 8a,b).

## Discussion

In this study, we implemented several parameters to the cell model, but varied only one of the four parameters, ACTIVATION THRESHOLD, DRAGGING TIME, SINGLE MOVING DISTANCE, and GAP PREFERENCE, for each cell type in each simulation; all other parameters were kept the same for all cells in the simulation.

In our first series of simulations, the ACTIVATION THRESHOLD parameter was varied (Figs. 4, 5). The parameter ACTIVATION THRESHOLD reflects a threshold dividing the two distinct modes of cellular behavior, *in stay *and *in move*. Recent fine quantifications of cell spreading and other motile activities have illustrated multiple phases of cellular behavior and thresholds for phase transitions [38-40]. Therefore, our assumption of a threshold for cell-cell interactions is not inappropriate; although the thresholds for the phase transitions observed in these previous reports were also correlated with cell-matrix interactions.

When several parameter sets with different values of ACTIVATION THRESHOLD for each cell type were examined, the "inside and outside" configuration of cell-sorting could not be reproduced. Since the high/low levels of ACTIVATION THRESHOLD reflect the low/high sensitivities to external signals, resulting in the low/high frequencies of the onset of cell movement, respectively, the frequency of the onset of cell movement may not be essential for the "inside and outside" configuration of cell-sorting. These results appeared to be inconsistent with previous findings that differences in the ACTIVATION THRESHOLD caused the "inside and outside" configuration of cell-sorting [41]. In this previous study, however, a cell was regarded as occupying a single lattice on a hexagonal lattice space. When a cell became *in move*, the cell always had to change position with a neighboring cell. In other words, the cell movement was discrete, and the distance of one cell movement was equal to the diameter of the cell causing the opposite cell movement of the neighboring cell. On the other hand, the present model in this study simulates quasi-continuous cell movement, which resembles the cellular behaviors *in vivo *more than those in the previous model. Thus, even when a cell became *in move*, the cell might not actually have been able to move, depending on its surroundings. Although the primary cause of the discrepancies between these two studies remains unclear, the above-mentioned difference in the method of cell movement may have been a critical factor.

When the DRAGGING TIME of the light cell type was ≤ 10 and that of the dark cell type was equal to 30, the "inside and outside" configuration of cell-sorting occurred when the ACTIVATION THRESHOLD equaled to 0.4, the SINGLE MOVING DISTANCE equaled to 1, and the GAP PREFERENCE was Htr (Fig. 6). When this pattern of cell sorting occurred, the light cells with the shorter DRAGGING TIME always surrounded a cluster of the dark cells with the longer DRAGGING TIME. Therefore, the cell type with the higher mobility "covered" the cell type with the lower mobility. In terms of molecular cell biology, the DRAGGING TIME parameter might correspond to the time required to reconstruct the cell body at a new core position through, for example, remodeling of the actin cytoskeleton [42] and the microtubule cytoskeleton [43] in a coordinated manner [44-46] and rear cytoskeletal contractility [47,48] during cell migration.

When the SINGLE MOVING DISTANCE values of the light cell type and the dark cell type were set at 2 and 1, respectively, the dark cells became localized near the center of the aggregate and the light cells surrounded the cluster of dark cells when the ACTIVATION THRESHOLD equaled to 0.4, the DRAGGING TIME equaled to 30, and the GAP PREFERENCE was Htr (Fig. 7). This result is consistent with the results of the simulations in which the DRAGGING TIME parameter was varied (Fig. 6), since SINGLE MOVING DISTANCE reflects the change in position of a cell during a single activation and thus should be negatively correlated with DRAGGING TIME. Indeed, the migration speed is known to be correlated with the retraction of the rear cell body in some cell types [47,48].

DRAGGING TIME and SINGLE MOVING DISTANCE exert direct influences on the way cells move, although ACTIVATION THRESHOLD provides only opportunities for cell movement. Thus, differences in mobility are important for the "inside and outside" configuration of cell-sorting, while differences in the frequency of the onset of cell movement may not be critically involved, as mentioned in the third paragraph of this section. Therefore, the current model was capable of distinguishing the processes of cell migration more finely than our previous model [41].

While the three parameters mentioned above are related to cellular motility, the fourth parameter – GAP PREFERENCE – determines the direction of cell movement [49-52]. When the light and dark cell types had Htr and Hm preferences, respectively, the "inside and outside" configuration of cell sorting was attained when ACTIVATION THRESHOLD equaled to 0.4, DRAGGING TIME equaled to 10, and SINGLE MOVING DISTANCE equaled to 1 (Fig. 8). Interestingly, the light cells became localized in the central region and the dark cells surrounded the cluster of the light cells.

These results suggest that 1) when two cell types with the Htr preference have different mobility from each other, or 2) when one cell type has the Hm preference and the other has the Htr preference, the "inside and outside" pattern of cell sorting arises. Cells with the Htr preference move so that they push the different cell type away, keeping the contact with the same cell type. Cells with the Hm preference move so that they penetrate between the same cell types as themselves. Within the aggregate, both cell types gathered compactly in a mutually exclusive manner. This cell "packing" reflects, as mentioned above, that both the Htr and Hm gap preferences commonly included specific adhesion properties.

Although the "inside and outside" pattern is important, as it is reminiscent of the original embryonic structures, the SAH model could not reasonably explain the "inside and outside" pattern as described in the Background section [9]. This has been the major weakness of the SAH model. Our present model overcomes this weakness in the SAH because it successfully recapitulated the "inside and outside" pattern based on the SAH.

On the other hand, the DAH model can also explain the "inside and outside" pattern. When one homophilic adhesive relationship (for example, between A and A) is strongest, the other homophilic adhesive relationship (for example, between B and B) is weakest, and a heterophilic adhesion (between A and B) is intermediate, the DAH predicts that these conditions are sufficient to generate the "inside and outside" configuration of cell-sorting [10]. Although early versions of the DAH model could not reproduce the "inside and outside" pattern, the Glaizer's model, at last, reproduced the "inside and outside" pattern excellently based on the DAH [23,24]. However, when both homophilic adhesions are stronger than the heterophilic adhesion, which corresponds to the "specific adhesive relationship", the DAH model predicts that the "inside and outside" configuration of cell-sorting does not occur. In the above Glazer's study (1992, 1993), this "specific adhesive relationship" was not examined. Thus, we reproduced their model (Additional files 1, 2), examined the final configurations of cell-sorting, and confirmed that the "inside and outside" configuration was not attained under the "specific adhesive relationship" with the DAH model (Additional File 3).

Although our model illustrated that the "inside and outside" pattern of cell-sorting is generated under a specific adhesive relationship, vectorial cellular movement in response to a cell's surroundings is indispensable and reflects a part of the characteristics of specific adhesion. We assumed that practically all the cells in multicellular organisms utilize this autonomous cell motility for morphogenesis during development. Morphogenesis is so dynamic that passive mechanisms such as static affinities and surface tension, are likely to be insufficient. Autonomous cell movement is common in the modeling of the slime mold *Dictyostelium discoideum*, since chemotaxis seen in *D. discoideum *is obvious as autonomous cell movement. In the theoretical study of *D. discoideum*, a diffusive signal is important and intercellular signaling is generally neglected. On the contrary, our model includes intercellular signaling and excludes diffusive signaling. Thus, our model and the models of *D. discoideum *are complementary for the modeling of morphogenesis during development.

Then, what molecular mechanisms could play an important role in the autonomous cell movement in our model? Members of the cadherin family are well known as specific cell-cell adhesion molecules [53,54] and may be the candidates supporting the cell behavior that was predicted in this study. Cadherins are known to play important roles in various developmental processes [55-58]. One of the functions of cadherins is the control of cell motility [57,59-62], which may be consistent with the findings obtained in this study. Moreover, cells with different exogenous cadherin expression patterns undergo cell-sorting [63]. Takeichi (1991) proposed a mechanism of intercellular signaling via the activity of cadherins during morphogenesis [64]. Niessen and Gumbiner (2002) showed that neither the static affinities of the extracellular domains of cadherins nor the adhesion of cells expressing one type of cadherin to sheets of extracellular domains of each cadherin determined cadherin-mediated cell sorting. Rather, the interactions between cells expressing each type of cadherin were important for the cell sorting [65]. Recently, several studies have shown that cadherin-mediated cell adhesion regulates actin assembly [66,67] and microtubule assembly [68] via Rho family molecules, which are major small GTPases important for cytoskeletal dynamics [69-71]. Conversely, the Rho family regulates cadherin-mediated cell adhesion [72,73]. IQGAP1, an effector of the Rho family, mediates Rho GTPases' regulation of cadherin-mediated cell adhesion [74,75]. These findings suggest a dynamic feedback loop between cell-cell adhesion and the cytoskeletons through the activities of Rho GTPases and their associated proteins, such as IQGAP1. Moreover, p120 catenin, which associates with the cytoplasmic domain of cadherins, might play an important role in regulating cadherin flow at the cell surface via microtubules [76] and endocytosis [77,78]. The endocytosis of cadherins is also regulated by Rho GTPases through IQGAP1 [79], and p120 regulates the activity of the Rho family [80]. Taken together, these observations suggest that cadherin-mediated cell adhesion and cytoskeletal remodeling compose a dynamic regulatory circuit for cell migration upon cell-cell contact. This conjecture resembles the molecular mechanism of integrin-mediated cell migration that depends on cell-cell matrix contacts [81,82] and may also fit our model. Although the molecular and cellular bases of the Htr and Hm preferences will need to be clarified in the future to eventually explain the mechanism underlying the 'inside and outside' pattern of cell sorting, the sufficient conditions predicted in this study may give new insight into the process of cell sorting.

Recently, constructing a comprehensive model of the intracellular dynamics using genome, proteome, and metabolome information is a trend in systems biology, because the high-throughput experiments enabled us to accumulate large amount of experimental data. Since our model is described as motile cellular automata, their inner states can easily be superimposed with the intracellular dynamics models like E-CELL[83]. By combining our model with the comprehensive models of intracellular dynamics, the systems biology approach may be expanded into inter- and multi-cellular level.

## Conclusion

This study suggests that the "inside and outside" configuration of cell-sorting can be successfully explained based on the concept of directional cell movement and the SAH. In addition, our results strongly suggest that the cell type which would ultimately be located near the center of the aggregate might be determined by the manner of directional movement and the mobility assigned to each cell type. These results can bridge findings at molecular or cellular level and mechanisms of morphogenesis during development. Since our model embodies various cellular behaviors that should be important for pattern formation, this model may also be applicable to other developmental processes beyond cell sorting.

## Methods

### Outline of *in silico *cell model

To simplify our model, a two-dimensional finite hexagonal lattice space is postulated. In this model, each cell occupies multiple hexagons in the lattice space (Fig. 1a), with the number of hexagons occupied by a single cell representing the cell's size. The standard cell size was set at 19 hexagons to allow for dynamic change in the cell's shape in response to its simulated surroundings. Each cell contains one hexagon that the cell never loses; this hexagon represents the cell's "core position." Whenever a cell is smaller than the standard cell size (= 19 hexagons), the cell attempts to occupy a vacant hexagon nearest to its core position. The vacant hexagon must be adjacent to hexagons occupied by the cell and must be located within a certain distance (= 4 hexagons) from the core position. Whenever a cell is larger than the standard size, the cell abandons the hexagon furthest from its core position. If abandoning the hexagon would cause a division in the cell, the cell retains that hexagon and abandons another hexagon. When this procedure is repeated for a single cell in a free lattice space, the cell tends to become round and of the standard size, with its core position located at the center of the cell (Fig. 1a). Thus, the core position may be regarded as the position at which a cell tries to remain in place at any given time point.

In a cluster of cells, the cells interact with one another and cause positional conflicts. If an expanding cell cannot find a suitable vacant hexagon, the cell attempts to occupy an already occupied adjacent hexagon nearest to its core position. When a cell occupies a hexagon formerly occupied by a neighboring cell, the neighboring cell loses that hexagon. This behavior represents the pushing of a neighboring cell. However, a cell cannot occupy a target hexagon if the hexagon is too close to the core position of the neighboring cell occupying that hexagon. This distance was set at 2 hexagons. Thus, this parameter models the pushing of a cell back against its neighboring cell, since the acquisition of the target hexagon would create an excessive invasion into the neighboring cell's body. In this situation, the cell then searches for another hexagon to acquire.

When a cell abandons one of its own hexagons, an adjacent cell often occupies that hexagon even if the size of the adjacent cell becomes larger than the standard size. This procedure represents the adjacent cell being stretched by mutual cell adhesion (Fig. 1b).

Because of positional conflicts, the individual cells in a cluster are often distorted, and the core position of a cell is not always at its center, defined as the hexagon with the minimum sum of distances from peripheral hexagons. If the shape of a cell is far from round (described later), the core position is shifted to an adjacent hexagon closer to the cell's center (Fig. 1c). This shift represents a cell being pushed away from its desired position.

During each step of the computer simulation, each cell performs the above-mentioned procedures, abandoning and acquiring new hexagons. If the cell becomes distorted, the procedure is re-performed. The acquisition or loss of a lattice as a result of a neighboring cell's behavior does not waste that cell's turn.

### Autonomous cell movement

We also simulated autonomous cellular motility. For convenience, two discrete cellular states were defined: *in stay *and *in move*. A cell *in stay *receives external signals from neighboring cells through its cell membrane and alters its inner state in response to those signals. The inner state of the *i*-th cell, *I*_*i*_, was defined as follows:

Ii=∑j=1NiΔIjkNi,
 MathType@MTEF@5@5@+=feaafiart1ev1aaatCvAUfKttLearuWrP9MDH5MBPbIqV92AaeXatLxBI9gBaebbnrfifHhDYfgasaacH8akY=wiFfYdH8Gipec8Eeeu0xXdbba9frFj0=OqFfea0dXdd9vqai=hGuQ8kuc9pgc9s8qqaq=dirpe0xb9q8qiLsFr0=vr0=vr0dc8meaabaqaciaacaGaaeqabaqabeGadaaakeaacqWGjbqsdaWgaaWcbaGaemyAaKgabeaakiabg2da9maalaaabaWaaabCaeaacqqHuoarcqWGjbqsdaqhaaWcbaGaemOAaOgabaGaem4AaSgaaaqaaiabdQgaQjabg2da9iabigdaXaqaaiabd6eaonaaBaaameaacqWGPbqAaeqaaaqdcqGHris5aaGcbaGaemOta40aaSbaaSqaaiabdMgaPbqabaaaaOGaeiilaWcaaa@41AF@

where *N*_*i *_is the number of lattice edges composing the border (= membranes) of the *i*-th cell and Δ*I*^*k*^_*j *_is the inner state increase determined by the signal of cell type *k *through the *j*-th edge of the border of the *i*-th cell.

When the inner state exceeds a threshold (ACTIVATION THRESHOLD, described later), the cell is activated and tries to move into a gap between adjacent cells. To move, the activated cell needs an adjacent cell that is *in stay *to use as a scaffold. If both adjacent cells composing a gap are moving, the gap is not penetrable. The activated cell's order of preference of penetrable gaps depends on the combinations of cell types by which the gaps are bound (described later). When a cell chooses a penetrable gap, the cell shifts its core position towards the chosen gap; thus, the cell moves in a direction from its core position and towards the chosen gap. On the other hand, the core position of the scaffold cell is simultaneously shifted in a direction opposite to the activated cell's direction of movement. Once the activated cell shifts its core position, the cell acquires an *in move *status and maintains its core position at the same position for several steps (described later); meanwhile, the scaffold cell is held by the moving cell and cannot move autonomously. Over the course of several steps, both the cell *in move *and the scaffold cell gather their occupied lattices around their respective core positions according to the acquisition or relinquishing of lattice hexagons described above. This means that the cell *in move *draws its body towards its core position while the scaffold cell is moved in the opposite direction as a physical reaction to the movement of the cell *in move *[84]. After several steps, the cell *in move *autonomously returns to an *in stay *status and releases the scaffold cell. When the activated cell cannot find suitable gaps, the activated cell becomes *in stay *and finishes its turn.

At each step, all cells perform their respective actions during their turn; the order of turns is decided randomly at the beginning of each step. A single simulation comprises 96,000 steps.

### Parameters regulating cellular behaviors

Several parameters reflecting cellular properties and behaviors were implemented: (1) the distortion score of the cell's shape; (2) the response to cell signaling promoting cellular movement; (3) the threshold of autonomous cell motility; (4) the order of preference of penetrable gaps, which determines the direction of cellular movement; (5) the duration of the *in move*-state; and (6) the distance of cell movement during a single activation.

The distortion score of a cell's shape was calculated as follows: the sum of the distances from the center of the cell to each lattice occupied by the cell was subtracted from the sum of the distances from the center of an imaginary round-shaped cell of the same size as the actual cell to each lattice of the imaginary cell and then divided by the cell's size. If the distortion score of a cell was larger than a parameter defined as MAXIMAL DISTORTION SCORE, the core position of the cell was shifted to an adjacent hexagon closer to the center of the cell's body. Thus, MAXIMAL DISTORTION SCORE represents the resistance of a cell to the pressure from neighboring cells.

To calculate the response to external cell signaling that promotes the movement of the cell in question, we assumed that Δ*I*^*s*^_*j *_= 0, Δ*I*^*d*^_*j *_= 1, and Δ*I*^*c*^_*j *_= Δ*I*^*m*^_*j *_= 2, (where *j *= 1...*N*_*i*_). The symbols *s*, *d*, *c*, and *m *denote the same cell type, different cell type, *cavity*, and *medium*, respectively. Both cavity and medium correspond to vacant hexagons: a vacant hexagon located within an aggregate was defined as a *cavity*, while a vacant hexagon located outside an aggregate was defined as a *medium*. Thus, signals from neighboring cells of the same cell type do not increase the *i*-th cell's inner state, while signals from different cell types increase the *i*-th cell's inner state and both cavity and medium classifications increase the *i*-th cell's inner state even more.

The threshold of autonomous cell motility was defined as ACTIVATION THRESHOLD. ACTIVATION THRESHOLD represents the resistance to contact with different cell types or alien stimuli (cavity and medium). If the inner state exceeds the ACTIVATION THRESHOLD, the *i*-th cell searches for penetrable gaps.

Five possible gaps were defined as follows: gaps between a) two cells of the same cell type as the cell in question, b) one cell of the same cell type as the cell in question and one cell of a different cell type, c) one cell of the same cell type as the cell in question and a cavity, d) one cell of a different cell type and a cavity, or e) two cells of different cell types. However, only gaps a), b), and c) were assumed to be penetrable, and gaps d) and e) were excluded. In other words, each cell moves in a manner that promotes contact with the same cell type. In view of the preference for cell type-specific contact and the no inner state increase by signals from the same cell type, our model generally reflects specific cell adhesion mechanisms.

After an activated cell chooses a gap and shifts its core position toward the chosen gap, the cell switches to an *in move *state, in which the cell attempts to keep its new core position at the new lattice unit and to gather its occupied hexagons around the new core position over the course of several steps that are parameterized as DRAGGING TIME. If the DRAGGING TIME is relatively long, the cell's shape stabilizes and the cell seems to remain in position in spite of its *in move *state. Since a cell with a short DRAGGING TIME quickly completes the reconstruction of its cell body, the cell can once again become ready to respond to external signals and to shift its core position to another new position. As a result, cells with a short DRAGGING TIME can move more quickly than cells with a long DRAGGING TIME under the influence of successive signals.

In addition to DRAGGING TIME, we defined another parameter for mobility: SINGLE MOVING DISTANCE. SINGLE MOVING DISTANCE defines the distance that the core position moves after a single activation. A cell with a longer SINGLE MOVING DISTANCE shifts its core position further than a cell with a shorter SINGLE MOVING DISTANCE when the cells have the same DRAGGING TIME.

### Initial conditions of randomly mixed aggregates

The finite hexagonal lattice space was set at 201 by 201. As the first step in the simulated cell rearrangement, we prepared a randomly mixed cell aggregate on the hexagonal lattice space as follows. The core positions of two cell types, 'light' and 'dark', were randomly placed at intervals of three lattices within a square area (73 ≤ x ≤ 127, 73 ≤ y ≤ 127). Since the initial cell size was set at 1, a period (3,000 steps) was assigned during which each cell expanded to attain a size of about 19 lattice units. None of the cells received external signals or moved autonomously during this period. The shape of the aggregate at the end of this period was roughly square (data not shown), but no signs of hysteresis as a result of the square shape were observed. The total number of steps in each simulation was 96,000, including the first 3,000 steps.

Usually, aggregation cultures of live cells are rotated to gather the cells together [85]. To mimic this force toward the center of the aggregate, the peripheral cells of the *in silico *aggregate were pushed toward the center of the hexagonal lattice space (100, 100) with a force reflecting the length of their membranes exposed to the medium. The force toward the center of the aggregate shifted the core positions of the cells from each current lattice to the neighboring lattice nearest to the center of the lattice space every four steps with a probability reflecting a ratio of the number of edges exposed to the medium to the number of edges of an imaginary round-shape cell of the standard size.

### Quantification and criteria of aggregate pattern

To classify the aggregation patterns, the areas within an aggregate and the periphery of the aggregate occupied by each cell type were measured using NIH ImageJ http://rsb.info.nih.gov/ij/ with the program's parameters set as follows: "distance in pixels" = 72, "known distance" = 1.00, "pixel aspect ratio" = 1.0, and "unit of length" = inch. The parameters were expediently set and did not reflect the actual scales.

To objectively determine whether the aggregates had attained an "inside and outside" configuration using each parameter set, we defined two parameters: CELL DISTRIBUTION RATIO and PERIMETER RATIO. The CELL DISTRIBUTION RATIO parameter indicates the ratio of the number of cells of a given cell type in the central region of the aggregate, which is divided into two regions (central and peripheral) with the same area, to the number of cells in the peripheral region. The PERIMETER RATIO parameter indicates the ratio of the length of the perimeter of an aggregate occupied by a given cell type to the length of the perimeter occupied by the other cell type. Based on our preliminary results, we classified the parameter sets as follows: when the mean CELL DISTRIBUTION RATIO of either cell type exceeded 3.0 and the mean PERIMETER RATIO of that cell type did not exceed 0.15 for a given parameter set, then the set was regarded as recapitulating the "inside and outside" configuration of cell-sorting.

## Competing interests

The author(s) declares that there are no competing interests. 

## Authors' contributions

TTM designed the cell model, performed simulation, and wrote the major part of the paper. KN and IA advised on the model and the simulation strategy based on the live cell observations and on the analysis of the results. KN directed and designed the entire project and is responsible for the paper. All authors have read and approved the final manuscript.

## Supplementary Material

Additional file 1 **Reproduction of results of Glazier's model 1**. As for parameters, see Glazier et al., 1993. (a) Initial configuration of homogeneous cell population. (b) Rounded pattern after 500 MCS: J_ll _= 2, J_lM _= 8, T = 5, and λ = 1. (c) 2 MCS annealing. Click here for file

Additional file 2 **Reproduction of results of Glazier's model 2**. As for parameters, see Glazier et al., 1993. (a) Checkerboard pattern after 3000 MCS: J_ll _= 10, J_dd _= 8, J_ld _= 6, J_lM _= J_dM _= 12, T = 10, and λ = 1. (b) Cell sorting after 20000 MCS: J_ll _= 14, J_dd _= 2, J_ld _= 11, J_lM _= J_dM _= 16, T = 10, and λ = 1. Click here for file

Additional file 3 **Results of Glazier's model under specific adhesions**. The following two parameter sets mean "specific adhesive relationship". As for parameters, see Glazier et al., 1993. (a) An example after 20000 MCS with conditions that J_ll _= 6, J_dd _= 4, J_ld _= 10, J_lM _= J_dM _= 16, T = 10, and λ = 1. (b) An example after 20000 MCS with conditions that J_ll _= 6, J_dd _= 4, J_ld _= 14, J_lM _= J_dM _= 16, T = 10, and λ = 1. Click here for file
