# Supplementary figures and images for: Computational cell model based on autonomous cell movement regulated by cell-cell signalling successfully recapitulates the "inside and outside" pattern of cell sorting
Source: BMC Syst Biol. 2007 Sep 20;1:43. doi: 10.1186/1752-0509-1-43 (PMC2100066; doi:10.1186/1752-0509-1-43)

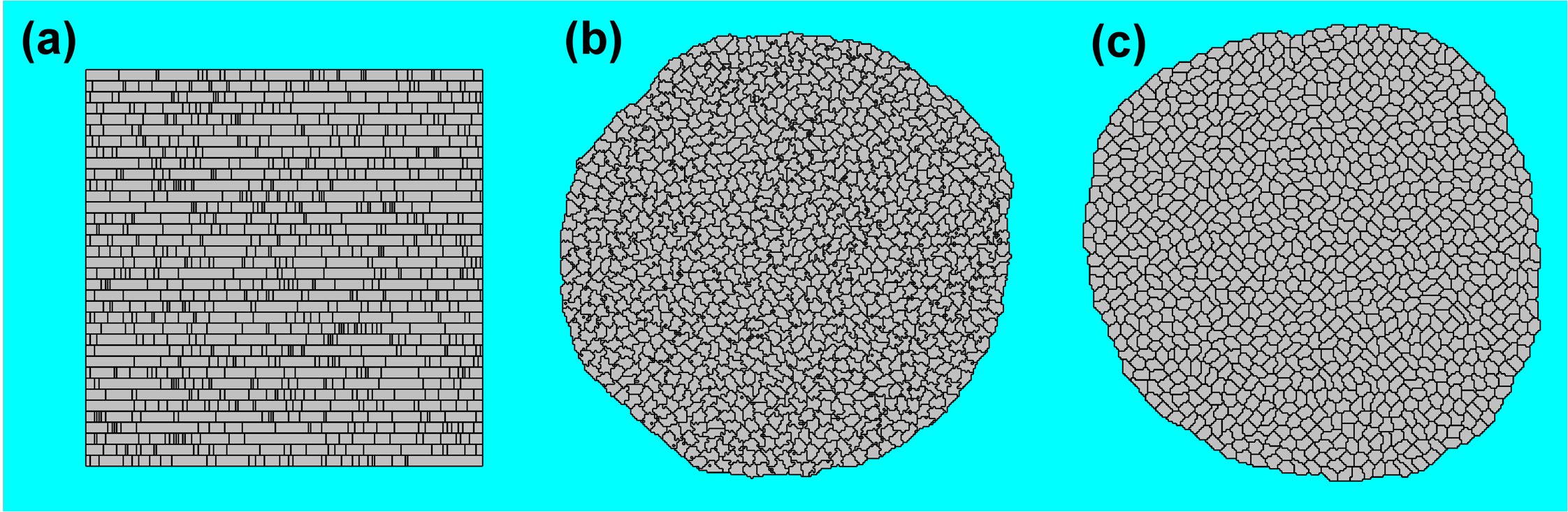

Supplement: Additional file 1 — Reproduction of results of Glazier's model 1. As for parameters, see Glazier et al., 1993. (a) Initial configuration of homogeneous cell population. (b) Rounded pattern after 500 MCS: Jll = 2, JlM = 8, T = 5, and λ = 1. (c) 2 MCS annealing. [file 1752-0509-1-43-S1.tiff]

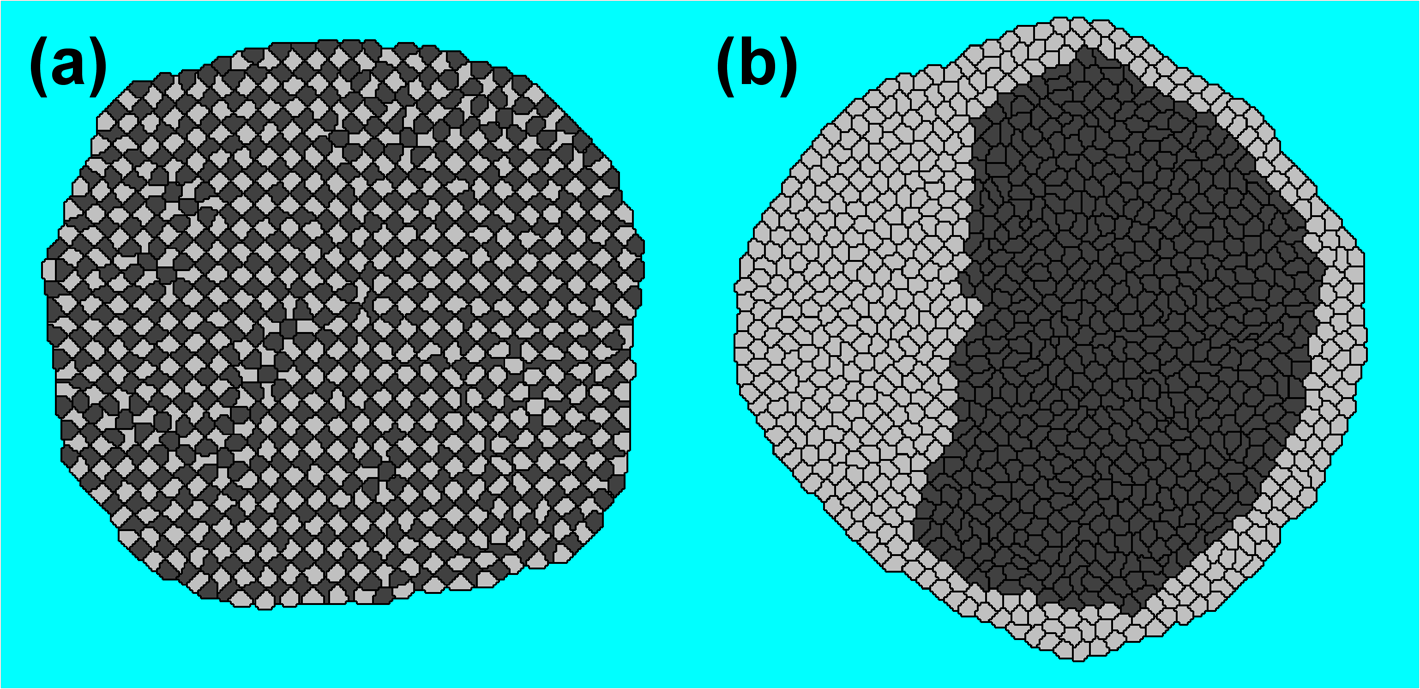

Supplement: Additional file 2 — Reproduction of results of Glazier's model 2. As for parameters, see Glazier et al., 1993. (a) Checkerboard pattern after 3000 MCS: Jll = 10, Jdd = 8, Jld = 6, JlM = JdM = 12, T = 10, and λ = 1. (b) Cell sorting after 20000 MCS: Jll = 14, Jdd = 2, Jld = 11, JlM = JdM = 16, T = 10, and λ = 1. [file 1752-0509-1-43-S2.tiff]

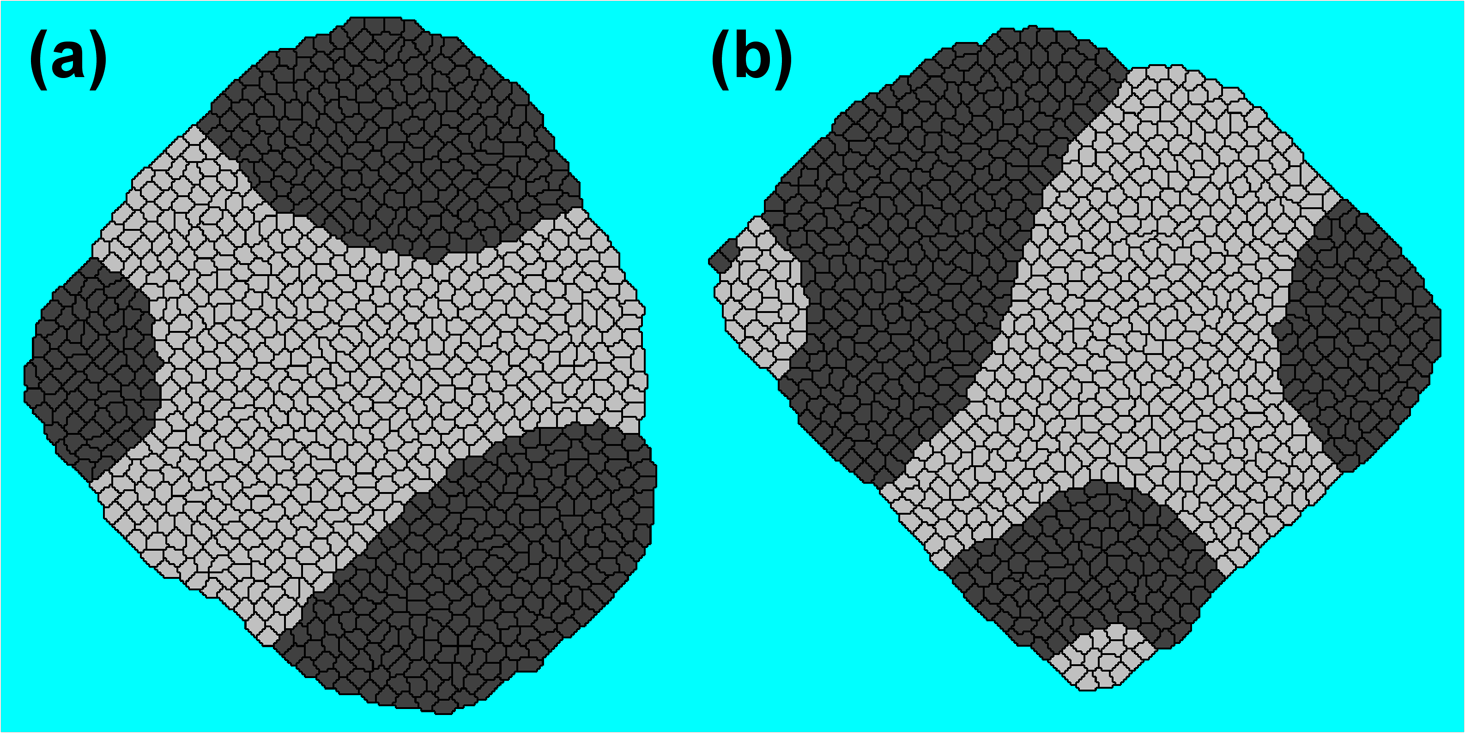

Supplement: Additional file 3 — Results of Glazier's model under specific adhesions. The following two parameter sets mean "specific adhesive relationship". As for parameters, see Glazier et al., 1993. (a) An example after 20000 MCS with conditions that Jll = 6, Jdd = 4, Jld = 10, JlM = JdM = 16, T = 10, and λ = 1. (b) An example after 20000 MCS with conditions that Jll = 6, Jdd = 4, Jld = 14, JlM = JdM = 16, T = 10, and λ = 1. [file 1752-0509-1-43-S3.tiff]
